# Verbal-analytical rather than visuo-spatial Raven's puzzle solving favors Raven's-like puzzle generation

**DOI:** 10.3389/fpsyg.2023.1205056

**Published:** 2023-11-17

**Authors:** Priyanka Srivastava, Saskia Jaarsveld, Kishan Sangani

**Affiliations:** ^1^Perception and Cognition Lab, Cognitive Science Center, Kohli Research Center on Intelligent Systems, Hyderabad, India; ^2^Cognitive and Developmental Psychology Unit, Centre for Cognitive Science, Kaiserslautern, Germany

**Keywords:** visuo-spatial, verbal-analytical, creative reasoning task (CRT), abstract reasoning, Raven's matrices, cognitive strategies, convergent and divergent thinking

## Abstract

Raven's advanced progressive matrices (APM) comprise two types of representational codes, namely visuo-spatial and verbal-analytical, that are used to solve APM puzzles. Studies using analytical, behavioral, and imaging methods have supported the multidimensional perspectives of APM puzzles. The visuo-spatial code is expected to recruit operations more responsive to the visual perception tasks. In contrast, the verbal-analytical code is expected to use operations more responsive to the logical reasoning task and may entail different cognitive strategies. Acknowledging different representational codes used in APM puzzle-solving is critical for a better understanding of APM's performance and their relationship with other tasks, especially creative reasoning. We used the eye-tracking method to investigate the role of two representational codes, visuo-spatial and verbal-analytical, in strategies involved in solving APM puzzles and in generating an APM-like puzzle by using a creative-reasoning task (CRT). Participants took longer time to complete the verbal-analytical than visuo-spatial puzzles. In addition, visuo-analytical than visual-spatial puzzles showed higher progressive and regressive saccade counts, suggesting the use of more response elimination than constructive matching strategies employed while solving verbal-analytical than visuo-spatial puzzles. We observed higher CRT scores when it followed verbal-analytical (*Mdn* = 84) than visuo-spatial (*Mdn* = 73) APM puzzles, suggesting puzzle-solving specific strategies affect puzzle-creating task performance. The advantage of verbal-analytical over visuo-spatial puzzle-solving has been discussed in light of shared cognitive processing between APM puzzle-solving and APM-like puzzle-creating task performance.

## Introduction

Intelligence and creativity are central to human beings and hold merit in one's success in performing simple to complex tasks and in achievements in one's life. Although intelligence appears to be a strong predictor of educational and occupational attainments, it often fails to predict creative achievements (Jauk et al., 2013). What constitutes intelligence and creative thinking and how these two constructs are related have been part of a longstanding debate. Despite decades of investigation, we still lack clarity on understanding these two constructs' relationships.

Intelligence and creativity have been considered to operate as a set (Sternberg and O'Hara, 2000; Jauk et al., 2013). Both sets have been considered either as coincident sets and treated as fundamentally identical or disjoint sets and treated as unrelated constructs or can be seen as a subset of each other with the overlap of features between the sets (Sternberg and O'Hara, 2000; Jauk et al., 2013). For instance, when creativity is considered a part of intelligence, it is described as one of the three intelligence measures along with analytical and practical intelligence (Gottfredson, 2003). In another example, creativity is considered a superset and is assumed to encompass intelligence either as the six necessary factors to achieve creativity (Sternberg and Lubart, 1995; Sternberg and O'Hara, 2000).

Whether intelligence is super or a subset of creativity, both types of models assumed a substantial correlation between the two constructs. However, the relationship was not observed to be linear in nature (see Jauk et al., 2013; Welter et al., 2016; Shi et al., 2017). Studies observed a positive correlation between intelligence and creativity with lower and average IQ scores. However, no correlation was observed with high IQ scores. It was argued that above-average intelligence (>120) could be considered a necessary but not a sufficient criterion for high creativity production (as creativity measures), and later known as a *threshold hypothesis* (Runco and Albert, 1986; Jauk et al., 2013). High IQ could result in high and low creative productions (Guilford and Christensen, 1973). Whereas, little evidence has been reported to support the high creativity scores with low IQ (Shi et al., 2017), indicating the necessary requirement of intelligence to perform creative tasks. Nevertheless, no consensus has been achieved in explaining the nature of cognitive requirements, or feature sharing between intelligence and creativity task performances (Jaarsveld and Lachmann, 2017; Eymann et al., 2022). These inconsistencies could be a result of methodological disparities and are pronounced by the way intelligence and creativity tests have been approached.

The intelligence tests include Raven's Progressive Matrices (Raven et al., 1998), Wechsler's Adult Intelligence Scale-Revised Vocabulary (WAISR-V) (Lee et al., 2014), and creativity tests used in these studies could be either or a combination of any of these tests: Torrance test for creative thinking (TTCT) (Torrance, 1972), test for creative thinking and drawing performance (TCT-DP) (Jellen and Urban, 1985), Guilford's alternative uses task (Wilson et al., 1953), and remote association test (RAT) (Lee et al., 2014). Intelligence tests are designed to measure convergent thinking, whereas creativity tests are designed exclusively to measure divergent thinking, except RAT. The RAT creativity test is the only test that measures the convergent thinking component of creativity; however, it does not express the convergent thinking component in its score (Lee et al., 2014).

The two types of thinking (i.e., convergent and divergent thinking) necessitate two different kinds of cognitive processes. Convergent thinking is associated with more focused cognitive control, focused and sustained attention, and an inhibitory control mechanism to interpret rules, deduce inference, and filter out irrelevant information. However, divergent thinking is associated with more distributed attention, cognitive flexibility, and an ability to shift attention between multiple ideas/components/sets by juggling varying scopes of attention for successful problem-solving (Vartanian, 2009; Colzato et al., 2012; Diamond, 2013; Lee and Therriault, 2013). The separate assessments of convergent and divergent thinking manifested in intelligence and creativity tests, respectively, increase the gap between the two constructs. In the case of creativity, the evaluation of divergent thinking or convergent thinking (RAT) in isolation is a fundamental issue with the operational definition of the construct (Jaarsveld and Lachmann, 2017) and restricts us from realizing that creativity is a product of both convergent as well as divergent thinking (Jaarsveld et al., 2013, 2015; Jaarsveld and Lachmann, 2017).

In addition, the widely used creativity and intelligence tests also differ in three other features: definition of the constructs, problem space, and knowledge domain (Jaarsveld et al., 2015; Jaarsveld and Lachmann, 2017; Eymann et al., 2022). The definition of construct refers to the measures of the cognitive operations it claims to measure. The problem space refers to the degree of freedom that can be exercised while approaching the problem, and the knowledge domain refers to the cognitive content of the given task (Jaarsveld and Lachmann, 2017). For a better understanding of shared cognitive processing between the two constructs, it is advised to keep the knowledge domain as same as possible.

Jaarsveld et al. (2015) and Jaarsveld and Lachmann (2017) have proposed a test called the creative reasoning task (CRT) that shares the knowledge domain with the intelligence test, namely Raven's advanced progressive matrices (APM), and also allows evaluation of both the convergent and the divergent thinking in a single interface. In CRT, participants are asked to create Raven's like (APM-like) puzzle in a given blank 3 × 3 matrix sheet. In CRT, the ideation process entails divergent thinking, and the evaluation and realization of a product or idea components entail analytical thinking, namely convergent thinking. Jaarsveld et al. (2013), Jaarsveld and Lachmann (2017), using CRT along with two other tasks, APM and TCT-DP, showed significant correlations between the scores of the APM and the scores of the convergent thinking component of the CRT, and between the scores of the TCT-DP and the scores of the divergent thinking component of the CRT. These results indicate the involvement of both components during creative reasoning processes. Since test pairs differed on a single variable only, significant correlation coefficients are found to be more meaningful.

Despite the use of different problem spaces, the shared knowledge domain between CRT and APM allows a better examination of strategies and associated cognitive mechanisms underlying these two constructs (Jaarsveld and Lachmann, 2017; Eymann et al., 2022). As CRT demands abstract reasoning in open space, it shares a significant portion of convergent thinking with APM puzzles. Although Jaarsveld and Lachmann (2017) posit an important point on sharing knowledge domain between intelligence and creativity by using APM and CRT, they lack examining the role of different representations required to solve the APM puzzle in the CRT task performance (Jaarsveld et al., 2015; Jaarsveld and Lachmann, 2017; Eymann et al., 2022). The evaluation of cognitive strategies associated with APM representational code, i.e., visuo-spatial and verbal-analytical, may offer critical insights into CRT performances, which is the focus of the current study.

Studies (Carpenter et al., 1990; DeShon et al., 1995; Prabhakaran et al., 1997; Chen et al., 2017) examining the representational codes of Raven's advanced progressive matrices (APM, explained below in the Method section, under the materials sub-heading) have divided the 36 puzzles into four major categories: pure visuo-spatial (e.g., APM 11 and 12), pure verbal-analytic (e.g., APM 1 and 4), either (e.g., APM 5 and 6), or both (e.g., APM 19 and 25) categories. The puzzles in the “either” category, such as APM 5, could be solved using either visuo-spatial or verbal-analytical strategies. The puzzles in the “both” category, such as APM 19, could be solved only while employing both strategies (Carpenter et al., 1990; DeShon et al., 1995; Chen et al., 2017). The visuo-spatial puzzles require a Gestalt approach, whereas verbal-analytical puzzles require a more descriptive and rule-based approach.

Furthermore, studies (Prabhakaran et al., 1997; Chen et al., 2017) using the fMRI method reported differential cortical responses for visuo-spatial and verbal-analytical APM puzzle-solving. They observed higher activity in cortical regions responsive to feature perception, like left medial temporal gyri (MTG), while solving visuo-spatial puzzles. Whereas, the verbal-analytical puzzle-solving was associated with cortical regions responsive to feature integration, hypothesis testing, and cognitive control, such as angular gyri, along with the verbal region. However, a study using eye-tracking measurements (Vigneau et al., 2006) such as scanning movement, dwell time, and saccadic movement did not report any significant difference between cognitive strategies employed while solving visuo-spatial and verbal-analytical puzzles. Instead, the study reported individual differences suggesting higher and lower cognitive ability in approaching APM corresponding to varying toggling counts of the eye movements across different areas of interest (see Vigneau et al., 2006).

**Figure 1:**
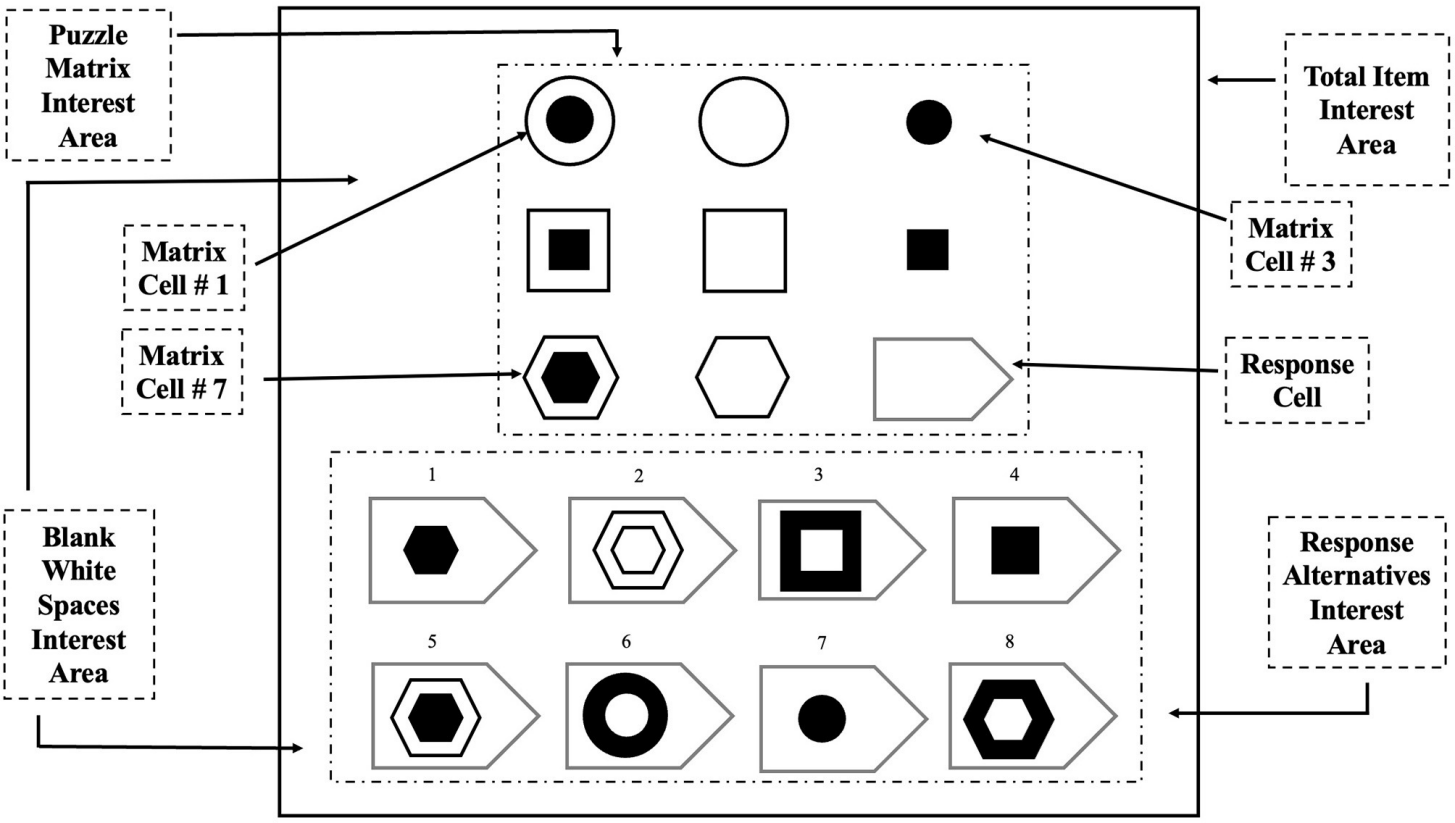
Hypothetical illustration of APM-like puzzle with eight alternatives and eye-tracking areas of interest in a given puzzle for the total item, puzzle matrix, response alternatives, and blank white space.

Vigneau et al. (2006) observed that participants with high APM scores used a more constructive matching strategy to analyze rules between matrix components and then compare the derived solution with the response alternatives for their final response selection. However, participants with low APM scores used a response elimination strategy to analyze rules between matrix components and make constant comparisons between the solution derivatives with the response alternatives before making their final response selection. The constructive matching strategy showed a comparatively lower count of toggling between the puzzle matrix area and the response alternatives area (Figure 1). The authors argued that the choice of strategy was more participant-specific than problem-specific (Vigneau et al., 2006).

Unlike previous study (Vigneau et al., 2006), the Cognitive Load Theory (Sweller, 1994, 2011) argues that it is the problem structure or the nature of the problem, not just the individual's cognitive ability, that determines the strategies for a given problem-solving. It could be possible that the APM puzzles with a higher number of rules and/or more abstract features might demand more response elimination strategy to reduce the working memory load, than a constructive matching strategy. The response elimination strategy would require more toggling between the puzzle matrix area and the response alternatives area to eliminate the inaccurate options for the given puzzle. Unlike Cognitive Load Theory, Vigneau et al. (2006) argued that individuals with lower working memory (WM) capacity might struggle with complex APM and therefore use the response elimination strategy. However, the study of Vigneau et al. (2006) did not conduct any working memory test to support their claim about individual-specific cognitive strategy. The distinct neural correlates of visuo-spatial and verbal-analytical puzzles (Prabhakaran et al., 1997; Chen et al., 2017) do not favor the Vigneau et al. (2006) finding, as the corresponding brain correlates were more problem-specific than individual-specific. In a nutshell, there appears a gap in understanding the relationship between the puzzle type and the required cognitive strategies to solve the puzzles.

### Purpose of the present study

The current study examines the effect of APM representational codes, i.e., visuo-spatial and verbal-analytical, on solving APM puzzles and creating an APM-like puzzle. We used eye-tracking measures to analyze the cognitive strategies associated with visuo-spatial and verbal-analytical APM puzzle-solving. We expect a better CRT performance when the CRT follows the verbal-analytical APM than visuo-spatial APM puzzles. The generation of APM-like puzzles using CRT share a more rule-based approach with verbal-analytical APM puzzle, and the visuo-spatial approach becomes more important only toward the end of the CRT process. At the beginning of the task, no visual material exists as yet. The visual material is generated by the participant during the design process. Only when there is material to be analyzed can the visuo-spatial approach contribute. Furthermore, we expect differential cognitive strategies while solving visuo-spatial and verbal-analytical APM puzzles.

We used multiple measures to evaluate the impact of the APM puzzle's representational code on CRT performance. The measures include the total time taken to solve puzzles, accuracy for solving APM puzzles, and count scores for CRT using CRT measures defined by Jaarsveld et al. (2012, 2015). We also asked participants' perspectives on the perception of complexity, effort, and engagement by using a brief survey taken from NASA Task Load Index (TLX, Hart and Staveland, 1988). We used the eye-tracking method to examine the strategies required to solve the visuo-spatial and verbal-analytical APM.

## Methods

### Participants

In total, 51 undergraduate and graduate students (41 males; mean age = 20.45 years, SD = 1.79) volunteered in the study. We excluded two participants' data because they did not meet the criteria of 50% accuracy of the given APM test. We used 50% accuracy criteria to ensure participants' engagement with the given puzzle type and induce the requisite approach associated with representational codes used in APM-solving. We used a total of 49 participants' data in our final analysis. All participants reported normal or corrected-to-normal vision. They were also screened using Snellen visual acuity (Hetherington, 1954) test by asking participants to read the letters until 20/20. Participants were monetarily credited for their contribution.

### Design

We chose a between-group experiment design to present the visuo-spatial and verbal-analytical APM puzzles separately to individual participants. A total of 49 participants were randomly allocated to the two independent conditions, namely, visuo-spatial and verbal-analytical APM puzzle-solving. The participants were naïve to the purpose of the experiment and performed the task individually and received identical instructions. We selected six APM puzzles labeled as pure visuo-spatial and six other puzzles labeled as pure verbal-analytical from Raven's advanced progressive matrices (APM). We made the selection based on the previous research, which showed differential behavioral and neural responses to these two types of puzzles (Carpenter et al., 1990; DeShon et al., 1995; Chen et al., 2017). The puzzle's properties are described in detail in the subsection APM puzzles under the Materials section.

### Apparatus

The experiment was conducted in an experimental lab at the university campus. We used a Dell 24′ monitor with 1,920 × 1,080 resolution, and Eye-link 1000 plus eye-tracking system for recording eye movement. Participants sat at ~80 cm from the monitor. We used the 13-gazing points detection accuracy with a threshold of 0.5 degrees or better. The saccades were detected using the default eye-link setup (velocity threshold set to 30°/s and acceleration threshold set to 8,000°/s2). An assistant experimenter sat behind the participant. The experiment was conducted in a dimly lit soundproof room.

#### Eye-tracking measures

We calculated the basic eye movements, such as saccade and fixation point, based on the standard procedure as described in SR Research Ltd (SR Research, 2017) (Eye-link II data viewer). With saccadic eye movements, we calculated total saccade, progressive saccade, and regressive saccade to understand the cognitive strategies employed for solving the visuo-spatial and verbal-analytical APM puzzle. We have described a more detailed analysis of the calculation procedure in the subsection Eye-tracking measures under the Results section.

### Materials

#### APM puzzles

The Raven's advanced progressive matrices (APM) is a standardized non-verbal fluid intelligence test and is widely used to assess an individual's non-verbal coherent and clear-thinking ability. The APM comprises 48 puzzles, presented in two sets: set I contains 12 puzzles and set II contains 36 puzzles. The APM puzzles chosen for this study were selected from set II.

We chose six pure visuo-spatial and six pure verbal-analytical puzzles from APM set II (DeShon et al., 1995; Chen et al., 2017). The six puzzles labeled visuo-spatial contained six types of relationships: movement, superimposition, addition/subtraction, superimposition with cancellation, mental transformation, and rotation (please refer to DeShon et al., 1995 for more details). In contrast, the six puzzles labeled as verbal-analytical contained three types of relationships: distribution of 2 or 3 with varying instances from 2 to 4, constant, and pairwise (please refer to DeShon et al., 1995 for more details). The order of six visuo-spatial and six verbal-analytical puzzles was kept identical to the standardized APM booklet. We selected only six puzzles to match the order of the standardized APM presentation across both groups. For instance, the 33rd puzzle labeled as visuo-spatial comprised rotation and superimposition with cancellation rule instance and was comparatively closely matched with the 34th vs. 36th puzzle labeled as verbal-analytical. The 34th puzzle comprised a distribution of three (three instances), whereas the 36th puzzle comprised a distribution of two (four instances) with constant rule instances. We chose the 34th compared to the 36th puzzle in the verbal-analytical puzzle set because of the proximity in progression with the 33rd visuo-spatial puzzle in Set II APM puzzles. The close comparisons between visuo-spatial and verbal-analytic were selected in respective orders: 3 and 4; 9 and 8; 12 and 13; 18 and 17; 22 and 21; and 33 and 34. The selected puzzles were presented under visuo-spatial and verbal-analytical conditions, and they were arranged in ascending order to match the standard APM display order. The visuo-spatial group contained 3, 9, 12, 18, 22, and 33 (DeShon et al., 1995). The verbal-analytic group contained 4, 8, 13, 17, 21, and 34 (DeShon et al., 1995). Puzzles that could not match the standard progression were not considered for this study.

Each APM Puzzle contains a 3X3 Matrix area and eight response alternatives, with only one correct response (Raven et al., 1998). The alternatives were presented below the puzzle (Figure 1). The puzzle in Figure 1 shows the relationship of “superimposition with cancellation” (DeShon et al., 1995), in which the overlapping borders and/ or features of given objects cancel each other out, and the final constructed object does not contain components common to the previous two objects. For example, in Figure 1, the third object in each row results from the cancellation of common components between the first and second object after superimposition by borders. All 12 puzzles were presented in black and white, subtending at a fixed visual angle.

#### Creative reasoning task (CRT)

The creative reasoning task (CRT) consists of an empty 3 × 3 matrix, similar to an APM puzzle, and participants were asked to create a Raven-like matrix in a given format (Please refer to Jaarsveld et al., 2012, 2015). Participants were asked to generate the complete puzzle, with the answer in the puzzle's last cell. In the present study, participants were not asked to generate response alternatives. We applied Jaarsveld's scoring method for relationships and components (Jaarsveld et al., 2013; Jaarsveld and Lachmann, 2017) for evaluating CRT performance.

#### Survey

The survey was taken after every puzzle to assess the perceived complexity of the given APM puzzle. The survey was based on NASA Task Load Index (TLX, Hart and Staveland, 1988), which evaluates individual subjective workload experience while performing any task or interacting with any system/machine. The current study used three measures of the NASA TLX, mental demand (experienced difficulty of the task), engagement, and effort. Each was measured on a 9-point Likert Scale (Table 1). We calculated the average score on each item of the survey.

**Table 1:** Complexity assessment questionnaire for each item.

**S.NO**.	**Complexity assessment**	**Likert scale (1 = very low, 5 = neutral, 9 = very high)**
1.	Difficulty—How difficult was the puzzle?	1 …… 5 …… 9
2.	Engagement—How engaging was the puzzle?	1 …… 5 …… 9
3.	Mental effort—How mentally demanding was the puzzle?	1 …… 5 …… 9

### Procedure

Each experimental session began with a consent form followed by instructions and Snellen's visual acuity test (Hetherington, 1954) to assess participants' visual acuity. Participants were given 3 warm-up APM puzzles from set I to get familiar with the settings, task, and mouse control. Each session was performed in a fixed order of APM items; and each APM item was displayed after a fixation screen, followed by the survey question (Table 1) regarding the puzzle. The fixation screen was presented for 4 s, and the puzzle and survey questions were presented until the corresponding responses were made. Participants were asked to perform the CRT (similar to Jaarsveld and Lachmann, 2017) after completing six APM puzzles as per their group conditions. The CRT start and end times were recorded by the experimenter, sitting at a distance. Participants were randomly allocated to either visuo-spatial or verbal-analytical conditions, which was followed by the CRT. To ensure that both APM conditions comprised puzzles with similar perceived complexity, we asked participants to self-report on complexity and effort after solving every APM puzzle (Table 1).

## Results

We selected a total of 12 puzzles from 36 APM set II puzzles. These 12 puzzles contained six visuo-spatial and six verbal-analytical puzzles as per DeShon et al. (1995) descriptions. The order of the presentation was kept identical to the standard APM puzzle set II. The correct response to the given APM puzzle scored “1”, and the incorrect response scored “0”. The cutoff was kept at 50% for further statistical analysis. In other words, only the data of those participants were selected for the analysis who could solve at least three out of six puzzles. One participant from visuo-spatial and one participant from verbal-analytical were not considered for the data analysis because either they showed <3 correct responses or no correct responses, respectively. Hence, we considered 49 participants' data, 24 visuo-spatial and 25 verbal-analytical, for the statistical analysis. The following sections will elaborate on APM and CRT analyses performed in this study. The Shapiro–Wilk test showed a violation of the normalcy assumption for all the measures, i.e., behavioral and eye-tracking, and therefore we chose non-parametric tests, namely Mann–Whitney *U*-test and Spearman's correlation, to analyze the data.

### APM task performance

The APM task performance was evaluated on three measures, total time taken (i.e., start and end time of a puzzle set, i.e., visuo-spatial and verbal-analytical), eye movement while performing the task, and self-report on perceived complexity: measuring difficulty, effort, and engagement after completing every puzzle. The eye movement was analyzed to understand the effect of visuo-spatial and verbal-analytical representation on cognitive strategies employed to solve the puzzle.

For eye-tracking analysis, the area of interest or region of interest was adapted from Vigneau et al. (2006). Each puzzle was divided into four major region of interests (RoIs): total item interest area, puzzle matrix interest area, response alternatives interest area, and blank white space interest area (Figure 1). The *total item* interest area contains the puzzle matrix interest area, response alternatives, and the blank spaces in between. The *puzzle matrix* interest area refers to the eight matrix cells of the 3 × 3 matrix along with the empty response cell. The *response alternatives* interest area refers to the eight response alternatives given below the puzzle matrix interest area. The *white blank space* interest area refers to the empty space in between the matrix interest area and response alternatives interest area, where no items are presented. As mentioned, we also analyzed the perceived complexity: difficulty, effort, and engagement of the given puzzle by employing a survey based on NASA TLX (Hart and Staveland, 1988), in which we asked the participant to rate the complexity of each puzzle, immediately after completing the puzzle.

In eye-tracking, we calculated dwell time, total saccade count, progressive and regressive saccade count, and scan path analysis to the defined RoIs (Figure 1). The dwell time represents the total amount of fixation time over RoI (Vansteenkiste et al., 2015). Ideally, fixation time helps us find the amount of time participants require to process some information. Longer fixation times imply participants' attention to the given area of interest that could be engaging (Theeuwes et al., 2004; Lagun and Lalmas, 2016) or complex and difficult to process the information (Jacob and Karn, 2003). Saccades are defined as the single rapid step-like movements between the peripheral region of interest, i.e., the direction of fovea sequential movement from one region of interest to another (Gegenfurtner, 2016). The progressive saccade represents the forward-directed saccadic eye movement (Liversedge and Findlay, 2000; Vigneau et al., 2006; Wan et al., 2020). For example, in Figure 1, in puzzle matrix RoI when the fixation transition occurs from one matrix cell to another matrix cell in either left-to-right (e.g., matrix cell 1 to matrix cell 3) or top-to-bottom (e.g., matrix cell 1 to matrix cell 7), it is counted as a progressive saccade. The regressive saccade represents the backward saccadic eye movement, i.e., moving backward through the RoIs (Liversedge and Findlay, 2000; Vigneau et al., 2006; Wan et al., 2020). For example, in Figure 1, in puzzle matrix RoI, when the eye fixation moves from one matrix cell to another matrix cell and then returns to the initial position either in left-right-left (e.g., matrix cell 1 to matrix cell 3 and returns to matrix cell 1) or top-bottom-top (e.g., matrix cell 1 to matrix cell 7 and returns to matrix cell 1), then it is counted as a regressive saccade. The total saccade is defined as the sum of progressive and regressive saccades.

#### Total time taken (seconds) to solve APM puzzles

**Figure 2:**
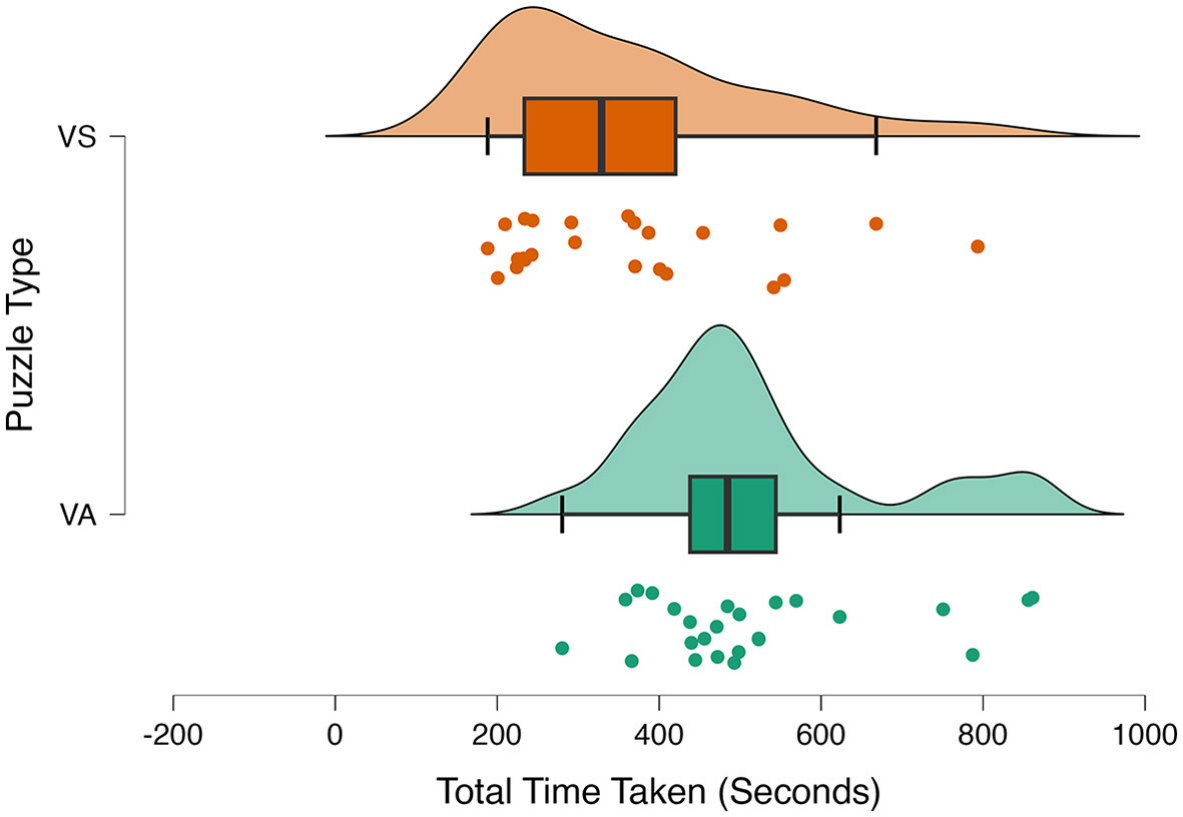
Raincloud plot for total time taken (seconds) to complete the APM puzzle under given conditions. VS, visuo-spatial; VA, verbal-analytical.

Two statistical analyses were performed to analyze the total time taken to solve the visuo-spatial and verbal-analytical puzzle set. We observed a violation of normality assumptions by using the Shapiro–Wilk test for both groups, visuo-spatial (W = 0.87, *p* = 0.006) and verbal-analytical (W = 0.88, *p* = 0.007). However, Levene's test for equality of variance was not statistically significant. We performed Mann–Whitney non-parametric test on the total time taken to solve visuo-spatial and verbal-analytical puzzles. The Mann–Whitney test indicated a significant main effect of the puzzle type on the speed of puzzle-solving with medium effect size (*U* = 133.00, *p* = *0*.001, *r* = −0.48). Participants took a longer time to solve the verbal-analytical puzzle set (*Mdn* = 484.49) than to solve the visual-spatial puzzle set (*Mdn* = 328.90) (Figure 2).

**Figure 3:**
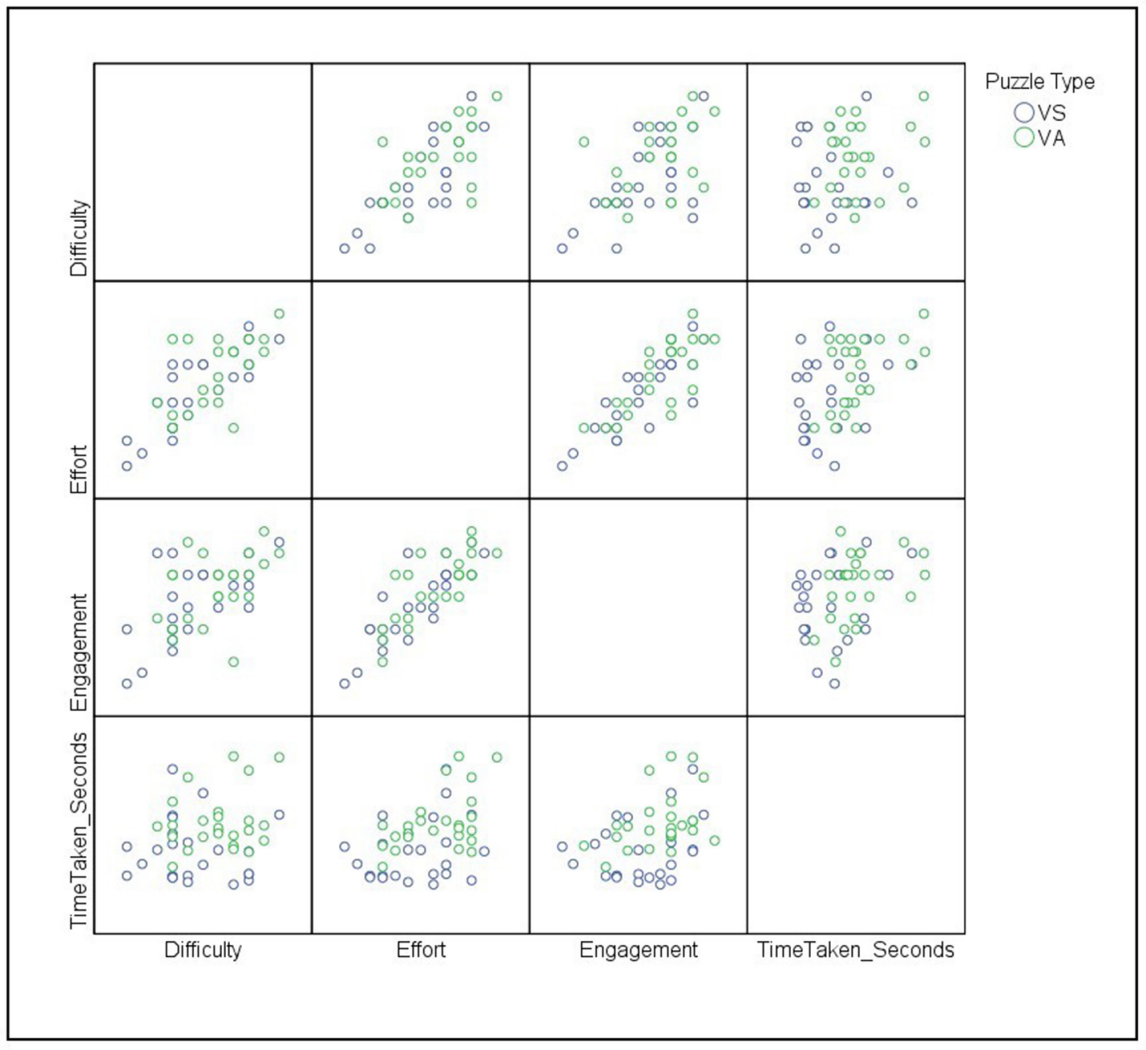
From the bottom left, the correlation variables are total time taken (seconds) and engagement, effort, and perceived difficulty in ascending order. We chose SPSS to construct the correlation across VS (visuo-spatial) and VA (verbal-analytical) conditions.

We selected Spearman's correlation coefficient to analyze the correlation between the total time taken to complete the puzzle under a given condition and self-reported puzzle complexity because we observed a violation of normality assumptions for total time taken for both conditions, VA and VS (reported above). We observed a moderately significant positive Spearman's correlation between the time taken to solve the puzzle and the self-reported engagement (*r* = 0.40*, p* = 0.004), and a moderately significant positive Spearman's correlation between the total time taken to solve the puzzle and self-reported effort (*r* = 0.40, *p* = 0.004) (Figure 3). We observed significant strong positive correlations between the three self-report items, indicating an internal consistency between items (Figure 3).

#### Eye-tracking measures while solving APM puzzles

The eye-tracking data did not follow the normal distribution pattern. The acquired data did not pass the Shapiro–Wilk's test of normality, except dwell time measures for response alternatives RoI, and saccades count for total item RoI. The violation of normalcy encouraged us to perform the Mann–Whitney *U*-test on the acquired eye-tracking data. We analyzed dwell time, total saccade count, progressive and regressive saccade, and scan path on a priori defined region of interests (RoIs), i.e., *total item matrix* RoI, *puzzle matrix* RoI, *response alternatives* RoI, and *blank white space* RoI (Figure 1).

##### Dwell time (seconds)

We have calculated the dwell time for all four regions of interest (RoIs) (Figure 1). The total item RoI dwell time represents the sum of fixations for all RoIs. The puzzle type showed a significant effect on the evaluation of total item RoI (*U* = 143*, p* = 0.002*, r* = −0.45) with comparatively higher dwell time with verbal-analytical (*Mdn* = 379.58 s) than visuo-spatial (*Mdn* = 254.32 s) puzzle set. Furthermore, a significant difference in dwell time was observed while evaluating the puzzle matrix RoI (Figure 1), with higher dwell time while analyzing verbal-analytical (*Mdn* = 237.92 s) than visuo-spatial puzzle set (*Mdn* = 143.01 s) (*U* = 155, *p* = 0.004*, r* = −0.41), and response alternatives RoI dwell time (Figure 1), with higher dwell time in verbal-analytical (*Mdn* = 88.41 s) than visuo-spatial (*Mdn* = 51.93 s) condition (*U* = 127, *p* < 0.001*, r* = −0.49).

We further calculated puzzle-wise dwell time for both puzzle matrix and response alternatives RoIs to evaluate the impact of self-reported difficulty, effort, and engagement on dwell time while analyzing visuo-spatial and verbal-analytical puzzles. Individual puzzle-wise dwell time analysis revealed an interesting result. Puzzle-wise *puzzle matrix* RoI (Figure 1) dwell time analysis showed significant differences between visuo-spatial and verbal-analytical puzzles with the first three puzzles, 3rd and 4th (*U* = 132, *p* < *0*.001, *r* = −0.48); 9th and 8th (*U* = 50, *p* < 0.001, *r* = −0.71); 12th and 13th (*U* = 85, *p* < *0*.01*, r* = −0.61). The later three puzzles under both representational code conditions did not show any significant effect on dwell time for puzzle matrix RoIs. For response alternatives RoIs, visuo-spatial and verbal-analytical puzzle-solving showed a significant effect on dwell time (Figure 1) while analyzing 9^t^h and 8th (*U* = 94.0, *p* < 0.001, *r* = −0.59); 12th and 13th (*U* = 84.00, *p* < 0.001, *r* = −0.62); and 33rd and 34th (*U* = 94.00, *p* < 0.001, *r* = −0.59) puzzles.

##### Saccade counts

Puzzle type showed a significant difference between verbal-analytical (*Mdn* = 1,447.0) and visuo-spatial (*Mdn* = 1,030.5) *total item* RoI saccade counts (*U* = 134.0, *p* = *0*.001, *r* = −0.47). In addition, puzzle type significantly influenced the progressive saccade count for *puzzle matrix* area (*U* = 193, *p* = 0.03, *r* = −0.30), and saccade counts for *blank space* (*U* = 125, *p* < 0.001, *r* = −0.5). Verbal-analytical puzzle type required more progressive saccade counts (*Mdn* = 159) than visuo-spatial (*Mdn* = 116) puzzle type, and participants made more saccade in blank space when solving verbal-analytical (*Mdn* = 993) than visuo-spatial (*Mdn* = 707.92) puzzle set.

Since regressive saccade represents the perceptive difficulty or more effort required to attend a given stimulus (Liversedge and Findlay, 2000; Vigneau et al., 2006), we further analyzed the regressive saccades as they could help understand the strategies involved in puzzle-solving. We observed a significant effect of puzzle type on regressive saccade at *puzzle matrix* interest area with higher regressive saccade while solving verbal-analytical (*Mdn* = 156.0) than solving visuo-spatial (*Mdn* = 106) (*U* = 191.50, *p* = 0.03*, r* = −0.31) puzzles. A similar effect was observed when saccades were made between *response alternatives* and *puzzle matrix* RoIs, with higher regressive saccade counts while solving verbal-analytical (*Mdn* = 38) than visuo-spatial (*Mdn* = 24.5) puzzles (*U* = 74.50, *p* < 0.001, *r* = −0.64). The results indicate that participants have employed different strategies while approaching two different types of puzzles.

The significant differences in self-reported difficulty, effort, and engagement at the first three visuo-spatial and verbal-analytical puzzles have encouraged us to analyze the progressive and regressive saccade counts at *puzzle matrix* RoI, and regressive saccade counts between *puzzle matrix and response alternatives* to evaluate the constructive matching and response elimination strategy while solving these two different kinds of puzzles. Progressive saccade counts at *puzzle matrix* RoIs showed a significant effect of puzzle type, with higher counts for verbal-analytical compared to visuo-spatial puzzles for the first three puzzles. The Mann–Whitney *U*-test statistics and median values are presented here in an order of visuo-spatial and verbal-analytical puzzles, 3rd (*Mdn* = 10) and 4th (*Mdn* =14) (*U* = 166, *p* = 0.007, *r* = −0.38); 9th (*Mdn* = 7.5) and 8th (*Mdn* = 23) (*U* = 62, *p* < 0.001*, r* = −0.68); and 12th (*Mdn* = 9.5) and 13th (*Mdn* = 18) *(U* = 96.50, *p* < 0.001, *r* = −0.58) puzzle sets. Similar trends were observed with regressive saccades count at the puzzle-wise *puzzle matrix* RoIs showing a significant effect of puzzle type, with higher counts for verbal-analytical compared to visuo-spatial puzzles for the first three puzzles. Puzzle-wise *puzzle matrix* RoI showed 3rd (*Mdn* = 9) and 4th (*Mdn* =13) (*U* = 162, *p* = 0.006, *r* = −*0*.39); 9th (*Mdn* = 7) and 8th (*Mdn* = 23) (*U* = 61.50, *p* < 0.001*, r* = −0.68); and 12th (*Mdn* = 9.5) and 13th (*Mdn* = 19) *(U* = 133.50, *p* = 0.001, *r* = −0.46) puzzle sets. Puzzle-wise regressive saccade counts between *puzzle matrix and response alternatives* RoIs showed a consistent trend except it did not show a significant difference at the first puzzles, and included 9th (*Mdn* = 2) and 8th (*Mdn* = 5) (*U* = 119.00, *p* < 0.001*, r* = −0.52), and 12th (*Mdn* = 2) and 13th (*Mdn* = 5) *(U* = 103.50, *p* < 0.001, *r* = −0.57) puzzle sets. Also, it did show a significant difference between the last puzzles, i.e., 33rd (*Mdn* = 4.5) and 34th (*Mdn* = 11) (*U* = 112*, p* < 0.001, *r* = −0.54) puzzle set, for visuo-spatial and verbal-analytical puzzles, respectively.

##### Scan path

The scan path analysis is the gazing pattern analysis of a participant for a selected region of interest (RoI) for a specific time period (Drusch et al., 2014; Eraslan et al., 2016). The scan path analysis revealed that participants go back and forth between puzzle matrix interest area and response alternatives during verbal analytical APM indicating a response elimination strategy. In the case of visuo-spatial APM, the participants spent a good amount of time in the puzzle matrix section itself and then shifted to the alternatives indicating a constructive matching strategy (Vigneau et al., 2006).

### Survey data

We collected self-reports from participants to assess the perceived complexity while approaching the visuo-spatial and verbal-analytical problems in terms of difficulty, effort, and engagement. There were six puzzles in each group of the APM. We observed significantly high self-reported difficulty and effort for the VA puzzles than VS, with *p* < 0.05. VA and VS did not show a significant difference in self-reported engagement response. We further looked at the puzzle-wise response to analyze the perceived complexity. The puzzle-wise examination showed a significantly higher self-reported difficulty and effort for the 4th, 8th, and 13th VA puzzles than for corresponding 3rd, 9th, and 12th VA puzzles. For difficult scores, the Mann–Whitney *U*-test statistical values are presented in order of VA and VS puzzles, 4th and 3rd (*U* = 202.00, *p* = 0.04, *r* = −0.288); 9th and 8th (*U* = 138.00, *p* < 0.001, *r* = −0.47); and 13th and 12th *(U* = 112.00, *p* < 0.001, *r* = −0.55) puzzle sets. For effort, the statistical values are presented in ascending order of VA and VS, 4th and 3rd (*U* = 198.00, *p* = 0.03, *r* = −0.30); 9th and 8th (*U* = 129.50, *p* < 0.001, *r* = −0.49); and 13th and 12th (*U* = 155.50, *p* = 0.003, *r* = −0.41) puzzle sets. However, the self-reported engagement was significantly high for only verbal-analytical 8th and 13th puzzles, in comparison to corresponding visuo-spatial 9th (*U* = 162.50, *p* = 0.005, *r* = −0.40) and 12th puzzles (*U* = 176.00, *p* = 0.01, *r* = −0.36).

### CRT score

**Figure 4:**
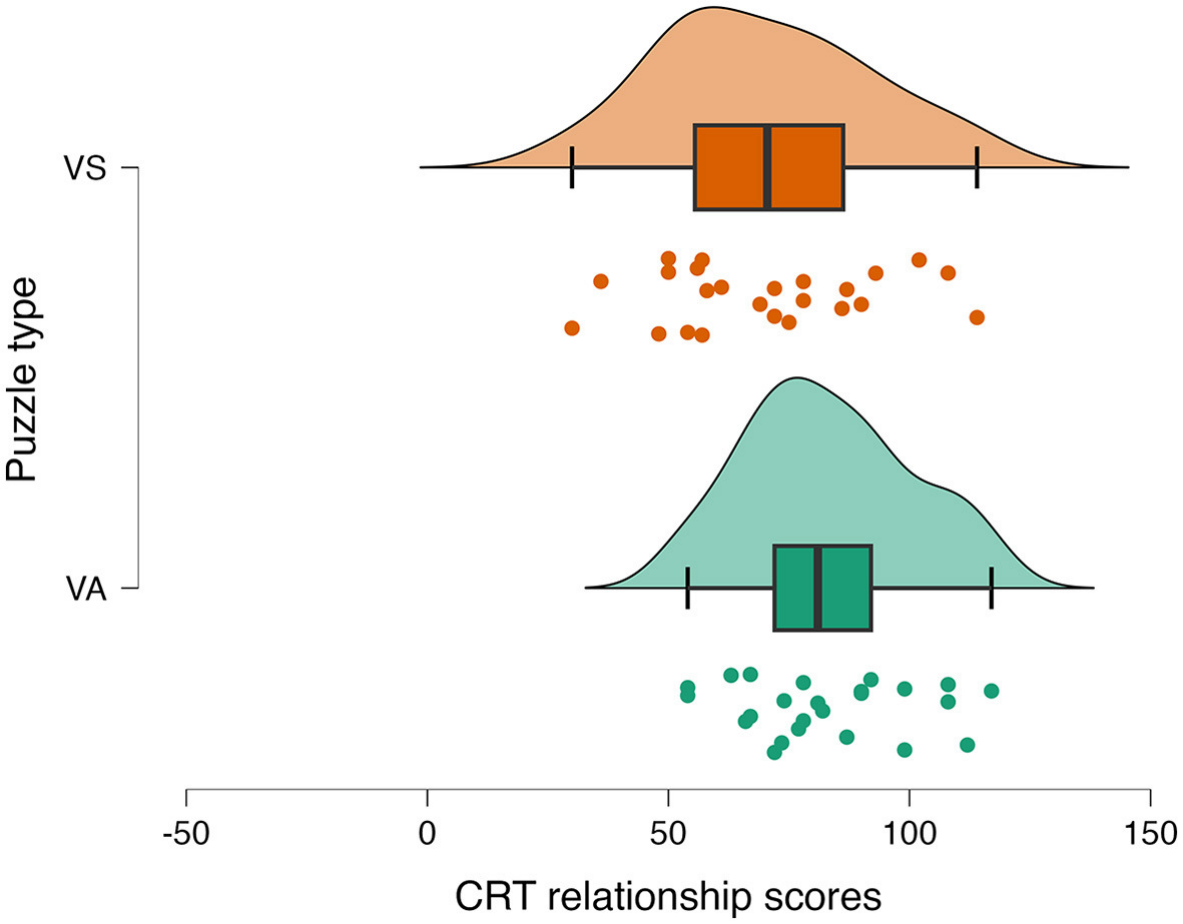
Raincloud plots for the total scores of CRT relations (rules) under two puzzle type conditions: VS (visuo-spatial) or VA (verbal-analytical) APM puzzles.

The CRT performance was scored as per Jaarsveld et al. (2012) and Jaarsveld et al. (2013) scoring method applied for CRT scoring. The CRT scores were calculated on two measures: components and relations. The relations represent mainly convergent thinking and the components represent divergent thinking (Jaarsveld et al., 2013). The CRT scores did not represent a normal distribution and led to choose non-parametric analysis. We have carried out the Mann–Whitney *U*-test on the total CRT scores of 49 participants. The results showed a significantly high CRT score when following the verbal analytical (*Mdn* = 84) than when following the visuo-spatial puzzle (*Mdn* = 73) (*U* = 187, *p* = 0.024*, r* = −0.32). We observed a significant effect of puzzle type on CRT relation score with high CRT relation score after solving verbal-analytical (*Mdn* = 81.0) than visuo-spatial (*Mdn* = 70.5) puzzle (*U* = 192, *p* = 0.03*, r* = −0.30). However, no significant difference was observed between CRT component scores when CRT was performed after verbal-analytical (*Mdn* = 3) and visuo-spatial (*Mdn* = 3) puzzles. The result suggests a differential effect of visuo-spatial and verbal-analytical progressive matrices on CRT reasoning performance (Figure 4). The result suggests that unlike visuo-spatial, verbal-analytical puzzle-solving strategy has a greater impact on convergent thinking of creative problem-solving task as verbal-analytical is more aligned with convergent thinking cognitive mechanism than a visuo-spatial puzzle-solving.

## Discussion

The current study was conducted to examine the effect of representation codes used in APM puzzle-solving (Raven and Raven's Progressive Matrices) on APM-like puzzle generation by using CRT (Jaarsveld et al., 2012). We chose CRT and APM tasks to keep the knowledge domain constant while varying the problem space, i.e., open and closed problem spaces, respectively, to reduce the other possible determinants for understanding the relationship between intelligence and creativity (Jaarsveld and Lachmann, 2017).

We made two novel observations. First, we observed a significant effect of APM puzzle type on APM-like puzzle formation measuring creative reasoning task performance. Participants showed a higher CRT score when CRT followed verbal-analytical APM puzzles compared to when CRT followed visuo-spatial APM. Second, unlike a previous study (Vigneau et al., 2006), the current study observed a difference in total time-on-task and eye-tracking measures according to APM puzzle types, visuo-spatial and verbal-analytical (DeShon et al., 1995). We will first discuss the role of visuo-spatial and verbal-analytical APM puzzles in cognitive processing, and then we will discuss the impact of visuo-spatial and verbal-analytical APM representation on CRT performance.

### Visuo-spatial and verbal-analytical APM

The difference in the total time taken to complete the verbal-analytical and visuo-spatial APM puzzles raises the question of whether this time difference is due to the strategical differences or the difference in complexity of the two types of puzzles, or both. We addressed these concerns in two stages. First, we matched the serial order of the visuo-spatial and verbal-analytical APM puzzles' presentation and kept them as comparable as possible (please refer to Materials section, APM puzzle). In the second stage, we asked our participants under both conditions to rate each presented puzzle at the level of self-perceived complexity, namely difficulty, effort, and engagement, immediately after completing their task.

The survey results showed statistically significant differences in the perceived level of difficulty, engagement, and effort across the two types of puzzles. However, the differences in self-reported difficulty and effort were observed only for the first three puzzle pairs (i.e., 3rd and 4th, 9th and 8th, and 12th and 13th, VS and VA puzzles, respectively). In the case of engagement, only two puzzles (9th and 8th, and 12th and 13th, VS and VA puzzles, respectively) showed significant differences. Furthermore, we analyzed the relationship between the total time taken to complete the puzzles and self-perceived task complexity, namely, difficulty, effort, and engagement. We observed no significant correlation between the total time taken to solve the puzzles and self-perceived difficulty. In contrast, a moderate correlation between self-reported engagement and total time taken to solve the APM puzzle and self-reported effort and time taken to solve the APM puzzle was observed.

The self-reported effort was supported by the total time taken to solve the two different kinds of APM puzzles and showed a consistent result. The difference in time taken to solve the puzzles under two different conditions suggests that two different strategies correspond to the differential effort required to solve the visuo-spatial and verbal-analytical puzzles. Unlike the previous study (Vigneau et al., 2006), the current study highlights the role of APM puzzle components in recruiting two different types of strategies than just individual differences.

Although the difference in self-reported complexity and the difference in total time taken to solve the visuo-spatial and verbal-analytical puzzle suggests a difference in effort required to apply a given problem-solving strategy, it does not provide any clarity on the evaluation processes involved. Is the difference in time also related to the evaluative process? The eye-tracking data helped understand the difference during puzzle evaluation while using two different cognitive strategies, namely response elimination and constructive matching strategies. The analysis of eye-tracking data showed a higher number of saccades count at puzzle matrix RoI and response alternatives RoI, a higher number of regressive saccades count between sub-components of puzzle matrix RoIs (matrix cell RoIs), a higher number of regressive saccades between puzzle matrix RoIs and response alternatives RoIs, and longer time spent on response alternatives RoIs for verbal-analytical matrices than visuo-spatial matrices. The higher number of saccades between the matrix interest area and response alternatives area and longer time spent on the response alternatives interest area have been associated with the “response elimination” strategy (Vigneau et al., 2006; Becker et al., 2016). However, longer time spent on matrix interest area and equal distribution of time across matrix cells have been associated with a “constructive matching strategy” (Vigneau et al., 2006; Becker et al., 2016). Unlike a previous study (Vigneau et al., 2006), the current eye-tracking results support the Cognitive Load Theory (Sweller, 1994, 2011), suggesting that it is the puzzle type or the nature of the puzzle and not the individual ability that determines the choice of problem-solving strategies.

The higher regressive saccade counts at puzzle matrix RoI, and between puzzle matrix and response alternative RoIs with verbal-analytical compared to visuo-spatial puzzles suggest that verbal-analytical puzzle entails response elimination, whereas visuo-spatial puzzles entail constructive matching strategy. The larger dwell time at response alternatives RoI when solving verbal-analytical compared to visuo-spatial puzzle, especially for the self-reported difficult and effortful puzzles, also support the interpretation of the response elimination strategy. However, the lower regressive saccade counts at puzzle matrix RoI, and the lower regressive saccade counts between puzzle matrix and response alternatives RoI, support more constructive matching strategy interpretation. Given that the first three verbal-analytical puzzles were perceived as more difficult and effortful, the longer dwell time is justified. In sum, the current results support the Cognitive Load Theory (Sweller, 1994, 2011) and suggest that the puzzle perceived as difficult and more effortful will demand more response elimination strategy than constructive strategy.

Previously, these two strategies, namely response elimination and constructive matching, have been argued in the context of individual differences in holding more information (working memory capacity) while finding a solution (Vigneau et al., 2006; Becker et al., 2016). Such findings suggest an individual-specific approach and argue that individuals are selective in their strategies and rarely use mixed types of strategy, indicating that these choices are individual-dependent and not problem-dependent (Becker et al., 2016). However, an alternative perspective, the “theory of cognitive load” proposed by Sweller (1994), suggests that the choice of a particular strategy might not only rely on individuals' preferences for problem-solving strategy or one's mental ability, but it might also depend on the nature of the problem. Using the theory of cognitive load (Sweller, 2011), we can argue for the possibility of a switch from a constructive matching strategy to a response elimination strategy if and when the problem demands. When a problem contains many elements or high interactivity between the elements, it demands a large working memory set and may require a response elimination strategy to enable more cognitively economical problem-solving than otherwise. Results from the current study indicate that the choice of strategy is more problem-specific than individual-specific. It is important to note that the difference in observed eye-tracking measures was limited to 50% of the puzzles as shown in puzzle-wise eye-tracking metrics analysis.

### APM and CRT

Raven's advanced progressive matrices may require different strategies adherent to visuo-spatial and verbal-analytical problems. However, the role of these strategies in creative reasoning tasks is not clear. Recently, studies (Mitchum and Kelley, 2010) have advocated for the constructive matching strategy to promote high skill and awareness rather than the use of a response elimination strategy. Unlike response elimination, the constructive matching strategy uses self-correcting feedback and enables performance monitoring and performance awareness. Advocacy for a particular strategy goes against the theory of cognitive load that argues in favor of the cognitive economy by eliminating the distractors for effective problem-solving. In addition, it is also not clear whether advocating a particular strategy would help approach creative problem-solving. The current study investigated the effect of problem-solving strategies induced by a given set of problems on creative reasoning. As mentioned above, the choice of response elimination strategy could result from complex problems to reduce the cognitive load for effective problem-solving. We assumed that if the response elimination strategy or constructive strategy is more problem-specific than individual-specific, then the choice for the problem-specific strategies might influence the creative reasoning task performances. If APM puzzle-solving is a more individual-specific task, then no difference in CRT task performance was expected.

Visuo-spatial reasoning entails operations that use more perceptual properties such as location, orientation, or direction. Whereas, verbal-analytical reasoning requires more abstract, analytical, logical operations to solve the given puzzle, such as category attribution. We hypothesized that if visuo-spatial and verbal-analytical reasoning entails two different cognitive processes, such as feature analysis and feature integration, respectively, and do use separate cognitive controls, these two processes may influence CRT performance deferentially. Like the APM puzzle, the CRT also requires cognitive processing that involves perception, cognitive control, and working memory. Since CRT requires both convergent and divergent thinking, it certainly demands high working memory capacity and high cognitive control to hold and manipulate the action plan to generate more rules between components used in creating APM-like puzzles. Given that verbal analytical APM puzzle-solving requires more working memory capacity and more cognitive control, it may prepare participants to perform CRT more effectively.

The current result supports the hypothesis that the multi-dimensional APM may affect CRT performance differently. The high CRT score following verbal-analytical than visuo-spatial APM supports the argument for choosing the problem-solving and problem-generation tasks that share certain cognitive processes while using two different problem spaces (Jaarsveld et al., 2015; Jaarsveld and Lachmann, 2017). When the two problems, problem-solving and problem-generation, are different in problem space but do share the knowledge domain, it allows better analysis of underlying cognitive processes. Since verbal-analytical APMs involve the more descriptive rule-based approach and require more working memory and attention control (Chen et al., 2017), the CRT performance was expected to be favored by the verbal-analytical APM than visuo-spatial APM. As CRT requires participants to think about generating rules, the verbal-analytical APM induces such reasoning and prepares the participants to construct the APM-like puzzle when they solve the visuo-spatial APM. The CRT *rule or relation score* expresses the number of rules applied between *components*, such as shape, used in creating APM-like puzzles. Participants who solved verbal-analytical APM scored higher on CRT creative reasoning compared to those who solved visuo-spatial APM. However, the *creative component* of CRT was not significant. The current result suggests that the CRT performance was affected by the strategy induced by solving the verbal-analytical and visuo-spatial APM items.

## Conclusion and future directions

The current study selected pure visuo-spatial and pure verbal-analytical puzzle types of the APM to investigate whether the choice of cognitive strategies in problem-solving is individual-specific or problem-specific and argued that if it is problem-specific then visuo-spatial compared to verbal-analytical problem-solving set will prepare an individual to approach the puzzle-creating task, CRT, differently. The difference in time taken to solve the two puzzle types and the difference in saccadic eye movement while approaching both types reinforce the idea that APM should be treated as a multi-dimensional rather than uni-dimensional structure. We suggest that APM scoring should incorporate this multi-dimensionality that represents two types of reasoning and may require two distinct cognitive processing. The difference in CRT scores, especially *relationships or rules* score, following verbal-analytical and visuo-spatial puzzle-solving tasks, supports the hypothesis that the puzzle-specific strategy influences the puzzle-creative task performance. The current results favor the argument for reducing the factorial complexities when investigating the relationship between intelligence and creativity by keeping the knowledge domain similar between the two constructs. In addition to the similarity in the knowledge domain, the current study recommends utilizing the cognitive overlap between well-structured and ill-structured problem-solving. The similarity in cognitive processes between well-structured and ill-structured problem-solving, especially those cognitive processes that enable functions like cognitive control, is expected to facilitate ill-structured problem-solving.

In the future, we need imaging studies to understand the role of shared cognitive processing in intelligence and creative thinking tasks. Additionally, we suggest focusing on the role of problem complexity in determining cognitive strategies alongside puzzle types. We recommend a factorial design among working memory, problem-specific cognitive strategy, and problem complexity to understand the relationship between individual working memory capacity and choice of problem strategies.

## Data availability statement

The raw data supporting the conclusions of this article will be made available by the authors, without undue reservation.

## Ethics statement

The studies involving humans were approved by Institute Review Board Committee, International Institute of Information Technology, Hyderabad. The studies were conducted in accordance with the local legislation and institutional requirements. The participants provided their written informed consent to participate in this study.

## Author contributions

PS: conception of the idea, hypothesis, experiment design, material selection, data collection protocols design, full manuscript writing with multiple revision of drafts, data analysis, data visualization, and data reporting. SJ: significant contribution to the conceptual development, theoretical feedback, and substantially supported manuscript writing and revisions. KS: primary technical writing, data collection, data coding, and preliminary data analysis. All authors provided critical feedback and helped shape the research, analysis, and manuscript. All authors contributed to the article and approved the submitted version.
